# Male sex and pretreatment weight loss are associated with poor outcome in patients with advanced non-small cell lung cancer treated with immunotherapy: a retrospective study

**DOI:** 10.1038/s41598-023-43866-5

**Published:** 2023-10-09

**Authors:** Jingxiao Jin, Jacqueline Visina, Timothy F. Burns, Brenda Diergaarde, Laura P. Stabile

**Affiliations:** 1 Department of Medicine, Division of Hematology-Oncology, University of Pittsburgh, Pittsburgh, PA USA; 2 Department of Internal Medicine, Vanderbilt University Medical Center, Nashville, TN USA; 3 UPMC Hillman Cancer Center, Pittsburgh, PA USA; 4 Department of Pharmacology & Chemical Biology, University of Pittsburgh, Pittsburgh, PA USA; 5 Department of Human Genetics, School of Public Health, University of Pittsburgh, Pittsburgh, PA USA

**Keywords:** Cancer therapy, Cancer, Immunology

## Abstract

The influence of sex and body mass index (BMI) on the efficacy of immune checkpoint inhibitors (ICIs) in advanced non-small cell lung cancer (NSCLC) patients remains unclear. We conducted a retrospective study to evaluate the relationship between sex, BMI, pretreatment weight loss (PWL), and clinical outcomes in 399 stage IV NSCLC patients treated with ICIs using data abstracted from medical records. Multivariable Cox proportional hazards models were used to assess the impact on overall survival and progression-free survival. Females were significantly more likely to experience immune-related adverse events and had a significantly lower risk of death compared to males in our patient cohort. In stratified analyses, the latter was limited to those receiving first-line monotherapy. BMI was overall not significantly associated with outcome. However, underweight patients had a significantly higher risk of both progression and death compared to normal weight patients in the first-line monotherapy group. When stratified by sex, underweight males had a significantly higher risk of progression and death compared to normal weight males. This was not observed among females. Those with PWL had overall significantly worse outcomes compared to those without. In stratified analyses, PWL was associated with significantly worse OS in both females and males. Stratified by treatment, the worse outcome was limited to those receiving ICI monotherapy. In summary, utilizing real-world data, this study suggests that male sex, being underweight, and PWL negatively impact ICI efficacy in NSCLC patients. Therapeutic approaches to improve ICI outcomes in underweight patients and those with PWL should be investigated.

## Introduction

Lung cancer is the leading cause of cancer death among both women and men in the United States^1^. Within the last decade, immune checkpoint inhibitors (ICIs) targeting programmed cell death 1 (PD-1), programmed cell death receptor ligand 1 (PD-L1) or cytotoxic T lymphocyte-associated antigen-4 (CTLA-4) have reshaped the treatment landscape for metastatic non-small cell lung cancer (NSCLC) patients^2^. These agents alone or combined with chemotherapy (chemoimmunotherapy) are now approved as first-line treatment for patients with advanced NSCLC without targetable driver mutations in epidermal growth factor receptor (*EGFR)* and anaplastic lymphoma kinase (*ALK)*^2^. Tumor PD-L1 expression is currently used to identify NSCLC patients eligible for treatment with ICI monotherapy^3^. Unfortunately, even among those with PD-L1 tumor proportion score (TPS) ≥ 50%, less than half of the patients respond to ICI monotherapy^4^. Adding chemotherapy to ICIs can improve the overall response rate (ORR) up to 50%. However, only 20–30% of patients achieve durable responses. As a result, the two year overall survival (OS) rate for NSCLC patients treated with ICIs is around 50% or less^2^. Therefore, better insight into the factors that predict response and prognosis is critical for the identification of those most likely to benefit from treatment with ICIs as well as for the development of novel immunotherapy approaches to improve outcomes in patients who do not currently benefit from ICIs.

While obesity is known to alter the tumor immune microenvironment (TIME) to accelerate tumor growth, it has also been linked to better outcomes in NSCLC patients treated with ICI monotherapy^5–9^. In a pooled analysis of four NSCLC clinical trials, obese (body mass index [BMI] ≥ 30 kg/m^2^) and overweight individuals (BMI 25–29.9 kg/m^2^) with PD-L1-positive tumors treated with atezolizumab monotherapy had longer OS compared to normal weight (BMI 18.5–24.9 kg/m^2^) PD-L1-positive patients^5^. The survival advantage of high BMI was not observed in the docetaxel-treated and PD-L1-negative groups. In addition, in a retrospective cohort of NSCLC patients with PD-L1 expression ≥ 50% treated with first-line pembrolizumab, obese patients had higher response rates and longer progression free survival (PFS) and OS compared to normal weight patients^8^. In a separate study, NSCLC patients with BMI ≥ 22 kg/m^2^ treated with ICIs in the second- or later-line setting had longer PFS and OS than those with lower BMI, although OS was not significant after controlling for potential confounders^9^. Conversely, a recent study on patients treated with chemoimmunotherapy reported no significant association of PFS and OS with baseline BMI^10^.

Early NSCLC studies conducted prior to the widespread use of immunotherapy also reported the association between higher BMI and improved survival, suggesting that obesity may be a favorable prognostic factor regardless of treatment modality^11, 12^. It is possible that the observed relationship is driven by underweight individuals experiencing cancer cachexia who were included in the lower BMI population. In line with this, recent studies have shown that in NSCLC patients receiving ICI monotherapy, cachexia (defined as unintentional pretreatment weight loss (PWL) greater than 5% during the six months prior to ICI initiation; some studies also include any degree of PWL > 2% and BMI < 20 kg/m^2^ or sarcopenia to this definition) was independently associated with lower response rates and shorter OS^13–15^. Similarly, PWL has been reported in some studies to be associated with worse outcome in those receiving chemoimmunotherapy^10, 16, 17^ Sarcopenia, or low muscle mass, may also contribute to poor ICI response. A recent study found that the protective effect of BMI on survival in melanoma and NSCLC patients disappeared after accounting for weight loss and skeletal muscle index^18^. However, other studies of skeletal muscle mass using cross-sectional imaging of computerized-tomography (CT) scans to predict ICI outcomes have yielded mixed results^19–22^.

It has been speculated that sex differences in muscle mass composition may be responsible for findings in melanoma demonstrating that males with higher BMI benefit more from ICIs than females with higher BMI^6, 23^. Furthermore, there is well documented sex-dimorphism in the immune system which could translate to a differential response to ICIs^24^. In support of sex differences in ICI response, a meta-analysis of early randomized controlled trials (RCTs) of ICIs *versus* standard of care chemotherapy in advanced solid tumors including melanoma and NSCLC reported that in males, ICIs had a significantly larger beneficial effect compared to standard of care than in women^25^. However, a subsequent meta-analysis found no significant association between patient sex and immunotherapy efficacy relative to standard of care regardless of cancer type, line of therapy, and immunotherapy agent^26^. Both of these studies included trials of immunotherapy alone and chemoimmunotherapy. A follow-up study reported that treatment with ICIs alone *versus* chemotherapy had a greater beneficial effect in males than in females, while females derived a larger benefit from treatment with chemoimmunotherapy^27^. However, these results were not replicated in subsequent retrospective analyses nor in a later meta-analysis that included more recent RCTs^28–30^. These findings underscore the complexity of sex-based effects on the immune system and response to ICIs.

While both sex and body composition can shape the immune composition of the TIME, the reported findings regarding sex- and body weight-dependent differences in response to immunotherapy are conflicting and highlight the need for additional investigations. In this observational study, we investigated the effects of sex, BMI and PWL on outcomes in metastatic NSCLC patients treated with ICIs at our institution, including a large proportion who received chemoimmunotherapy.

## Methods

### Study population

We conducted a retrospective study of 399 consecutive patients diagnosed with stage IV NSCLC and treated with ICIs between January 2017 and January 2020 (inclusive) at UPMC Hillman Cancer Center (Pittsburgh, PA). The electronic medical records (EMR) at our institution were scanned in April 2020 for patients with lung cancer older than 18 years of age and treated with a PD-1/PD-L1 or CTLA-4 agent, or other immunotherapy agent; this initially identified 880 patients. After detailed chart review, 481 patients were excluded based on the following criteria: not stage IV, received ICI outside of specified timeframe, ICI for malignancies other than NSCLC (including small cell lung cancer), previously treated with ICI in the early-stage setting (including durvalumab consolidation for stage III disease), and unavailable pathology or radiological reports.

Demographic and clinical data including age, sex, height, weight, serum creatinine and albumin levels, neutrophil–lymphocyte ratio (NLR), treatment and outcome information, and information on adverse events were abstracted from the EMR using a structured questionnaire and entered into a study-specific REDCap database. BMI was calculated as weight in kg divided by height in meters squared (kg/m^2^), and standard World Health Organization (WHO) definitions were used to create BMI categories: underweight (BMI < 18.5 kg/m^2^), normal weight (BMI 18.5–24.9 kg/m^2^), overweight (BMI 25–29.9 kg/m^2^), and obese (BMI ≥ 30 kg/m^2^). When available, weight six to twelve months prior to ICI initiation was also abstracted. This was used to determine PWL; a patient was classified as having PWL if they had lost 5% or more of their body weight in the six to twelve months prior to the initiation of ICI treatment. One of the criteria for cancer cachexia according to the international consensus definition is 5% loss of stable body weight over the past six months^31^. For our study, we expanded the time span criteria to include up to twelve months, as many patients did not have weight information available at exactly six months prior to treatment initiation. Treatment was categorized as first-line immunotherapy, non-first line immunotherapy, or chemoimmunotherapy. The first-line and non-first line immunotherapy groups include patients who received anti-PD-(L)1 inhibitors as monotherapy, as well as three patients who received combination nivolumab and ipilimumab. The chemoimmunotherapy group includes patients who received concurrent immunotherapy during first-line chemotherapy as well as patients who were rechallenged with chemoimmunotherapy two or more years after previous chemotherapy. Patients in this group did not previously receive immunotherapy monotherapy. The study was reviewed and approved by the University of Pittsburgh IRB.

### Response assessment

Determination of best response was based on treating oncologist’s documentation and interval imaging reports. Patients with no evidence of disease on restaging scans were considered to have a complete response (CR), while a decrease in the size of existing lesions was considered a partial response (PR). Minimal or no change in the size of existing lesions was considered stable disease (SD). If there was increase in the size of existing lesions on initial restaging scans, pseudoprogression was presumed, and the next set of scans was reviewed. On the second set of scans, if there was decreasing or minimal change in size of lesions, the patient was determined to have SD. Otherwise, they were considered to have progressive disease (PD), with the date of progression backdated to the date of the first set of restaging scans. Any new lesions, including oligometastatic central nervous system metastases, were considered evidence of PD. Disease control was defined as any best response other than PD: either CR, PR, or SD. PFS was calculated as the time from initiation of immunotherapy to progression of disease or date of last follow-up. OS was calculated as the time from initiation of immunotherapy to death from any cause or date of last follow-up.

### Immune-related adverse events

Occurrence of immune-related adverse events (irAEs), including dermatitis, hyperthyroidism, hypothyroidism, hypophysitis, pneumonitis, colitis, gastritis, hepatitis, nephritis, cerebritis, mucositis, immune thrombocytopenia, neuropathy, arthritis/arthralgias, and myositis/myalgias was determined through review of oncologist notes. IrAEs were categorized as mild (not requiring systemic steroids), requiring systemic steroids but not therapy limiting, or therapy limiting. Adverse events were determined to be related or unrelated to immunotherapy based on the clinical judgment of the treating oncologist.

### Statistical analyses

Chi-square tests were used to compare categorical demographic and clinical patient characteristics including best response (CR, PR and SD *vs*. PD) and presence of irAEs between sex, BMI and PWL categories; Wilcoxon–Mann–Whitney and Kruskal Wallis tests were used to compare continuous patient characteristics. Kaplan–Meier curves were estimated for OS and PFS; curves were compared using log-rank tests. The reverse Kaplan–Meier method was used to estimate the median follow-up time. Multivariable Cox proportional hazards models were used to assess the effect of sex (male was used as reference), BMI (as categorical variable; normal weight was used as reference), and PWL (as categorical variable; no PWL was used as reference) on OS and PFS; hazard ratios (HRs) and corresponding 95% confidence intervals (CIs) were calculated. Survival analyses were conducted overall as well as stratified by treatment line and sex. *P* values < 0.05 were considered significant. Statistical analyses were performed using SAS® (version 9.4, SAS Institute Inc., Cary, NC, USA).

### Ethical approval

This retrospective study was approved by the University of Pittsburgh Institutional Review Board committee.

## Results

### Patient characteristics

The study population consisted of in total 399 patients with metastatic NSCLC; selected characteristics are presented in Table 1. Mean age was 68.0 years, 49.9% of the patients were female and the majority was white and ever smoker. Adenocarcinoma was the most common histology (65.9%). PD-L1 TPS was > 50% in 117 (29.3%) of patients. Presence of an *EGFR* mutation was reported for 16 (4.0%) patients, none had an *ALK* translocation. *KRAS* mutation was identified in the tumor of 137 patients (34.3%), and of these 53 (38.9%) were *KRAS* p.G12C. At initiation of ICI therapy, 31 (7.8%) patients were underweight, 146 (36.6%) had normal weight, 124 (31.1%) were overweight, and 98 (24.5%) were obese. Information on PWL was available for 281 (70.4%) patients; among these, 138 (49.1%) lost 5% or more of their body weight in the six to twelve months prior to the initiation of ICI treatment. The majority of the patients received pembrolizumab and almost half were treated with chemoimmunotherapy. Almost 61% showed disease control (CR, PR or SD), and the majority (66.7%) had no reported irAEs. Overall median follow-up time was 24.2 months (95% CI 23.0–25.6), and 234 (58.6%) patients died during follow-up. Overall median PFS was 5.7 months (95% CI 5.2–6.6); overall median OS was 17.8 months (95% CI 14.3–20.1).

**Table 1:** Selected characteristics of the study population (N_total_ = 399).

	N (%)^a^
Age at IT^b^ initiation in yrs	
Mean (± sd)	68.0 (± 9.9)
Range	(33.1–92.6)
Sex, female	199 (49.9)
Race	
White	348 (87.2)
Black/African American	36 (9.0)
American Indian/Alaska Native	1 (0.3)
Asian	6 (1.5)
Unknown	8 (2.0)
Smoking status	
Never	23 (5.8)
Former	188 (47.4)
Current	186 (46.9)
Performance status (ECOG)	
0–1	301 (75.4)
2+	63 (15.8)
Unknown	35 (8.8)
Serum creatinine (mg/dL), mean (± sd)	0.94 (± 0.48)
Albumin (g/dL), mean (± sd)	3.54 (± 0.57)
Neutrophil–lymphocyte ratio, mean (± sd)	8.3 (± 10.6)
Body mass index at IT^b^ initiation in kg/m^2^	
Mean (± sd)	26.4 (± 6.2)
Range	(13.4–49.4)
Body mass index at IT^b^ initiation in categories	
Underweight (< 18.5 kg/m^2^)	31 (7.8%)
Normal weight (18.5–24.9 kg/m^2^)	146 (36.6%)
Overweight (25–29.9 kg/m^2^)	124 (31.1%)
Obese (≥ 30 kg/m^2^)	98 (24.5%)
Pretreatment weight loss^c^	
Yes	138 (34.6)
No	143 (35.8)
Unknown	118 (29.6)
Histology	
Adenocarcinoma	263 (65.9)
Squamous cell carcinoma	77 (19.3)
Other^d^	59 (14.8)
Metastatic stage	
M1a	130 (32.6)
M1b	60 (15.0)
M1c	209 (52.4)
PD-L1 status	
< 1%	146 (36.6)
1–50%	94 (23.6)
> 50%	117 (29.3)
Unknown	42 (10.5)
Mutation status	
* KRAS (all)*	137 (34.3%)
* KRAS G12C*	53 (13.3%)
* EGFR*	16 (4.0%)
* ALK*	0 (0%)
Immunotherapy received	
Pembrolizumab	283 (70.9)
Atezolizumab	71 (17.8)
Nivolumab^e^	45 (11.3)
Treatment line	
1st line monotherapy	101 (25.3)
Non-1st line monotherapy	108 (27.1)
Chemoimmunotherapy	190 (47.6)
Best response	
Disease control (CR, PR, or SD)^f^	242 (60.7)
Progressive disease (PD)	157 (39.4)
Immune-related adverse events (irAEs)	
No AE	266 (66.7)
AE mild	54 (13.5)
AE steroids^g^	30 (7.5)
AE limiting^h^	49 (12.3)
Alive at last follow up	165 (41.4)

^a^Does not always add up to N = 399 due to missing information.

^b^IT: immunotherapy.

^c^5% or more weight loss in the 6 months–1 year prior to immunotherapy initiation.

^d^Other: adenosquamous carcinoma (N = 3), large cell carcinoma (N = 1), large cell neuroendocrine (N = 17), poorly differentiated NSCLC (N = 13), high grade neuroendocrine carcinoma (N = 4), sarcomatoid carcinoma (N = 7), mixed (N = 8), unknown (N = 6).

^e^Includes three patients who received nivolumab plus ipilimumab.

^f^Complete response (CR), N = 4; partial response (PR), N = 141; stable disease (SD), N = 97.

^g^AE requiring steroid treatment but not therapy limiting.

^h^AE that was therapy limiting.

### Females had better overall survival compared to males

We first examined whether sex was associated with immunotherapy efficacy and toxicity. There was no significant difference in rate of disease control between females and males in our study population, but females more frequently had irAEs compared to males (39.2% *vs*. 27.5%, *P* = 0.01). (Table 2). Among those with irAEs, 50.0% of the females had mild adverse events *versus* 27.3% of the males. Females were also more often never smokers (9.1% *vs*. 2.5%, *P* = 0.01) and underweight (11.1% *vs*. 4.5%, *P* = 0.01). Females had significantly lower serum creatinine levels (0.83 ± 0.36 *vs*. 1.05 ± 0.55, *P* < 0.0001), but there were no significant differences in frequency of PWL, serum albumin levels and NLR between females and males (data not shown).

**Table 2:** Characteristics by sex (N_total_ = 399).

N(%)	Female (N = 199)	Male (N = 200)	*P*-value^a^
Age at IT^b^ initiation in yrs			0.50
Mean (± sd)	67.8 (± 10.1)	68.3 (± 9.8)	
Smoking status			0.01
Never	18 (9.1)	5 (2.5)	
Former	96 (48.5)	92 (46.2)	
Current	84 (42.4)	102 (51.3)	
Performance status (ECOG)			0.81
0–1	150 (75.4)	151 (75.5)	
2+	30 (15.1)	33 (16.5)	
Unknown	19 (9.6)	16 (8.0)	
Pretreatment weight loss^c^			0.17
Yes	77 (38.7)	61 (30.5)	
No	70 (35.2)	73 (36.5)	
Unknown	52 (26.1)	66 (33.0)	
PD-L1 status			0.13
< 1%	79 (39.7)	67 (33.5)	
1–50%	47 (23.6)	47 (23.5)	
> 50%	59 (29.7)	58 (29.0)	
Unknown	14 (7.0)	28 (14.0)	
Body mass index at IT^b^ initiation in categories			0.07
Underweight (< 18.5 kg/m^2^)	22 (11.1)	9 (4.5)	
Normal weight (18.5–24.9 kg/m^2^)	72 (36.2)	74 (37.0)	
Overweight (25–29.9 kg/m^2^)	55 (27.6)	69 (34.5)	
Obese (≥ 30 kg/m^2^)	50 (25.1)	48 (24.0)	
Body mass index at IT^b^ initiation in categories			0.01
Underweight (< 18.5 kg/m^2^)	22 (11.1)	9 (4.5)	
BMI ≥ 18.5 kg/m^2^	177 (88.9)	191 (95.5)	
Treatment line			0.81
1st line monotherapy	49 (24.6)	52 (29.1)	
Non-1st line monotherapy	52 (26.1)	56 (35.8)	
Chemoimmunotherapy	98 (49.3)	92 (35.1)	
Best response			0.28
Disease control (CR, PR, or SD)^d^	126 (63.3)	116 (58.0)	
Progressive disease (PD)	73 (36.7)	84 (42.0)	
Immune-related adverse events			0.01
Yes	78 (39.2)	55 (27.5)	
No	121 (60.8)	145 (72.5)	

^a^*P* value from chi-square test for categorical variables and from Wilcoxon–Mann–Whitney test for continuous variables.

^b^IT: immunotherapy.

^c^5% or more weight loss in the 6 months–1 year prior to immunotherapy initiation.

^d^CR: complete response; PR: partial response; SD: stable disease.

**Figure 1:**
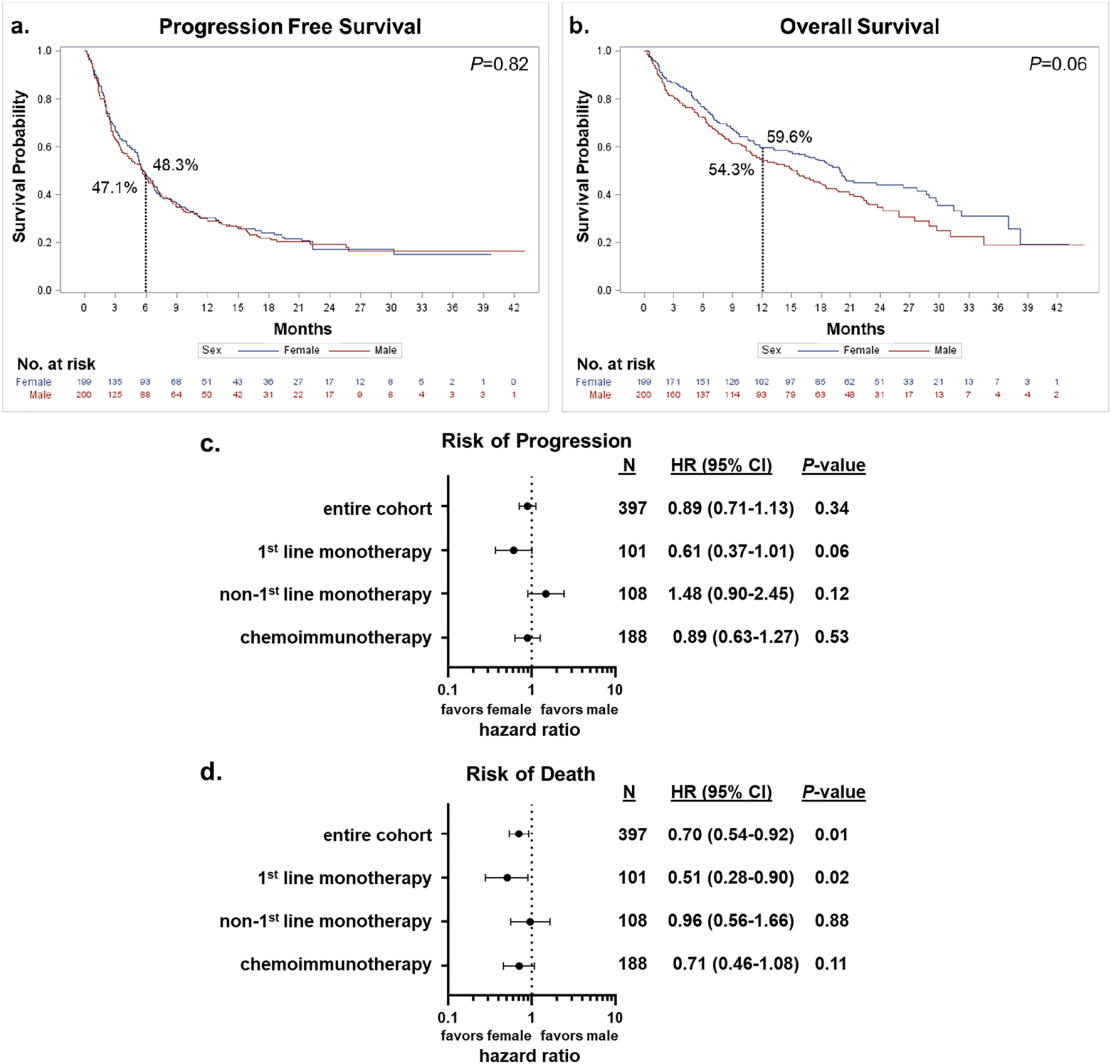
Sex and patient outcomes. (**a**) Kaplan–Meier progression free survival (PFS) estimates by sex. Six-month PFS rate is indicated. (**b**) Kaplan–Meier overall survival (OS) estimates by sex. Twelve-month OS rate is indicated. Curves were compared using log-rank tests. (**c**) and (**d**) are Forest plots reporting results from the multivariable analyses of the effect of sex on risk of disease progression and death, respectively. The reference group is “male”, adjusted hazard ratios (HRs), corresponding 95% confidence intervals (CIs) and *P* values are shown. Variables included in the Cox proportional hazards model: age at immunotherapy initiation, BMI at immunotherapy initiation (underweight, normal, overweight, obese), smoking status (never, former, current), ECOG performance status at immunotherapy initiation (0–1, 2 + , unknown), and pretreatment weight loss (no, yes, unknown).

Kaplan–Meier curves for PFS and OS by sex are presented in Fig. 1a,b. No significant differences in outcomes were observed between males and females in the entire cohort (Fig. 1a,b) or when stratified by treatment line (data not shown). Results from the multivariable analyses are reported in Fig. 1c,d (adjusted for age at immunotherapy initiation, BMI at immunotherapy initiation, smoking status, ECOG performance status at immunotherapy initiation, and PWL). We observed no significant sex differences with risk of disease progression but females had an overall lower risk of death compared to males (HR: 0.70, 95% CI 0.54–0.92, *P* = 0.01). Analyses stratified by treatment line showed a significantly lower risk of death (HR: 0.51, 95% CI 0.28–0.90, *P* = 0.02) for females compared to males among those who received first-line monotherapy. The association with disease progression was in the same direction but not significant (HR: 0.61, 95% CI 0.37–1.01, *P* = 0.06). No significant sex differences in risk of progression or death were observed among those on any other treatment line. Additional adjustment for PD-L1 status, irAEs status and creatinine level did not alter these results (data not shown).

### Underweight patients had worse outcomes

We next examined the relationship between BMI at initiation of immunotherapy and outcomes. Best response to treatment differed significantly between the four BMI categories with PD being most common among underweight patients (Table 3). No significant difference in response was observed between normal (34.2% PD), overweight (40.3% PD) and obese (36.6% PD) individuals (*P* = 0.58), but PD was significantly more common among underweight patients (BMI < 18.5 kg/m^2^; 67.7% PD) compared to those with BMI ≥ 18.5 kg/m^2^ (37.0% PD; *P* = 0.0008). Underweight patients were significantly more often female and more likely to have PWL than those with BMI ≥ 18.5 kg/m^2^ (71.0% *vs*. 48.1%, *P* = 0.01, and 61.3% *vs*. 32.3%, *P* = 0.002, respectively). In addition, underweight individuals also had significantly lower serum creatinine levels (0.66 ± 0.22 *vs*. 0.79 ± 49, *P* < 0.0001), and serum albumin levels (3.28 ± 0.70 *vs*. 3.56 ± 0.55, *P* = 0.01) than those with BMI ≥ 18.5 kg/m^2^; there was no significant difference in NLR (data not shown). PWL was least common among obese patients (BMI < 30 kg/m^2^ *vs*. ≥ 30 kg/m^2^: 39.5% *vs*. 19.4%, *P* = 0.001). The frequency of irAEs did not differ significantly between the four BMI categories.

**Table 3:** Characteristics by body mass index category (N_total_ = 399).

N(%)	Underweight^a^ (N = 31)	Normal weight (N = 146)	Overweight (N = 124)	Obese (N-98)	*P*-value^b^
Sex, female	22 (71.0)	72 (49.3)	55 (44.4)	50 (51.0)	0.07
Age at IT^c^ initiation in yrs					0.10
Mean (± sd)	64.4 (± 10.2)	69.0 (± 10.3)	68.6 (± 9.3)	67.1 (± 9.7)	
Smoking status					0.009
Never	2 (6.5)	6 (4.1)	7 (5.6)	8 (8.3)	
Former	9 (29.0)	58 (39.7)	71 (57.3)	50 (52.1)	
Current	20 (64.5)	82 (56.2)	46 (37.1)	38 (39.6)	
Performance status (ECOG)					0.08
0–1	19 (61.3)	108 (74.0)	103 (83.1)	71 (72.5)	
2 +	10 (32.3)	24 (16.4)	13 (10.5)	16 (16.3)	
Unknown	2 (6.5)	14 (9.6)	8 (6.5)	11 (11.2)	
Pretreatment weight loss^d^					< 0.0001
Yes	19 (61.3)	65 (44.5)	35 (28.2)	19 (19.4)	
No	4 (12.9)	38 (26.0)	55 (44.4)	46 (46.9)	
Unknown	8 (25.8)	43 (29.5)	34 (27.4)	33 (33.7)	
PD-L1 status					0.30
< 1%	13 (41.9)	54 (37.0)	45 (36.3)	34 (34.7)	
1–50%	7 (22.6)	36 (24.7)	21 (16.9)	30 (30.6)	
> 50%	9 (29.0)	44 (30.1)	38 (30.7)	26 (26.5)	
Unknown	2 (6.5)	12 (8.2)	20 (16.1)	8 (8.2)	
Treatment line					0.70
1st line monotherapy	9 (29.0)	39 (26.7)	27 (21.8)	26 (26.5)	
Non-1st line monotherapy	10 (32.3)	33 (22.6)	38 (30.7)	27 (27.6)	
Chemoimmunotherapy	12 (38.7)	74 (50.7)	59 (47.6)	45 (45.9)	
Best response					0.006
Disease control (CR, PR, or SD)^e^	10 (32.3)	96 (65.8)	74 (59.7)	62 (63.3)	
Progressive disease (PD)	21 (67.7)	50 (34.2)	50 (40.3)	36 (36.7)	
Immune-related adverse events					0.21
Yes	6 (19.4)	52 (35.6)	46 (37.1)	29 (29.6)	
No	25 (80.7)	94 (64.4)	78 (62.9)	69 (70.4)	

^a^Underweight (< 18.5 kg/m^2^), normal weight (18.5–24.9 kg/m^2^), overweight (25–29.9 kg/m^2^), and obese (≥ 30 kg/m^2^).

^b^*P* value from chi-square test for categorical variables and from Kruskal Wallis test for continuous variables.

^c^IT: immunotherapy.

^d^5% or more weight loss in the 6 months–1 year prior to immunotherapy initiation.

^e^CR, complete response; PR, partial response; SD, stable disease.

Kaplan–Meier curves for PFS and OS by BMI are presented in Supplemental Fig. 1. Overall, no significant differences in outcomes were observed between the four BMI categories (normal, underweight, overweight and obese; Supplemental Fig. 1a,b). Similarly, no significant differences in PFS and OS were observed when comparing BMI < 30 kg/m^2^ *vs*. BMI ≥ 30 kg/m^2^ (*P* = 0.78 and *P* = 0.80, respectively) and BMI < 18.5 kg/m^2^ *vs*. BMI ≥ 18.5 kg/m^2^ (*P* = 0.09 and *P* = 0.06, respectively). Stratified by treatment line, OS differed significantly between the BMI categories among patients treated with first-line monotherapy only (*P* = 0.04), with underweight patients doing the worst (12-month OS rate: 22.2% *vs*. 52.6%, 58.5% and 71.0% for normal, overweight and obese, respectively); the difference in PFS was not statistically significant (*P* = 0.06) (Supplemental Fig. 1c,d). Stratified by sex, PFS and OS differed significantly between the BMI categories among males (*P* < 0.0001 for both), with underweight patients doing the worst (6-month PFS rate: 0% *vs*. > 35.0% for the other BMI categories;12-month OS rate: 11.1% *vs*. > 50.0% for the other BMI categories), but not among females (Supplemental Fig. 1e,f).

**Figure 2:**
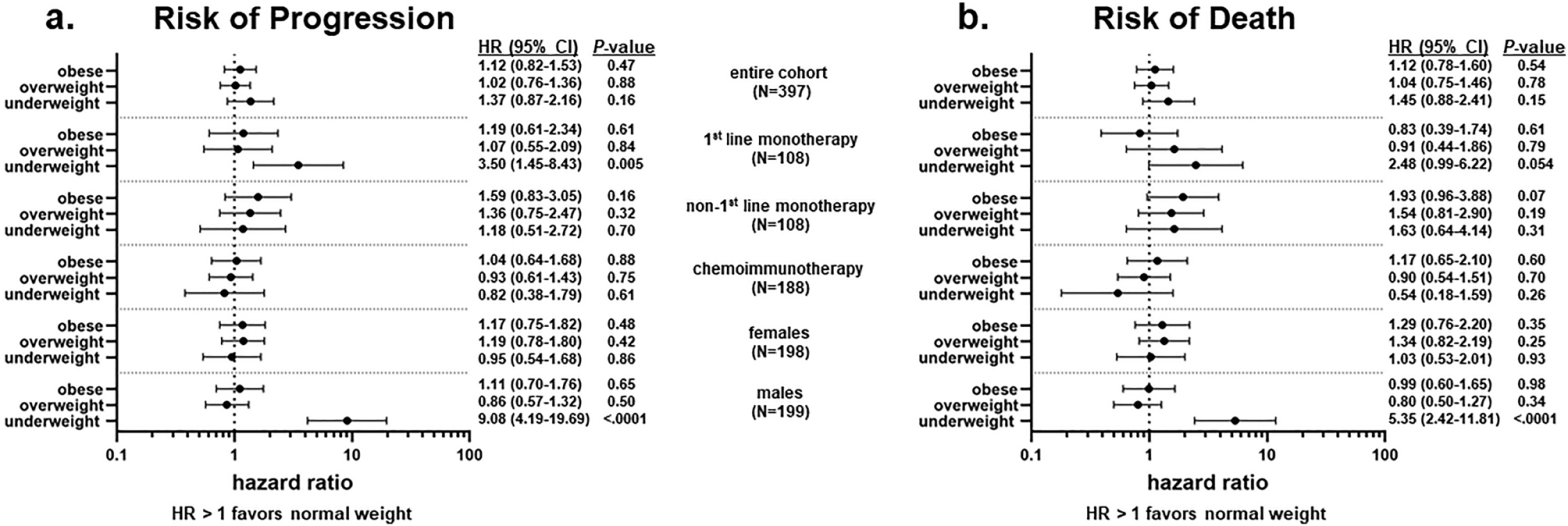
BMI and patient outcomes. Forest plots reporting results from the multivariable analyses of the effect of body mass index (BMI) on risk of disease progression (**a**) and death (**b**). The reference group is “normal weight”, adjusted hazard ratios (HRs), corresponding 95% confidence intervals (CIs) and *P* values are shown. Variables included in the Cox proportional hazards model: age at immunotherapy initiation, sex (female, male), smoking status (never, former, current), ECOG performance status at immunotherapy initiation (0–1, 2 +, unknown), and pretreatment weight loss (no, yes, unknown) (sex was not included in the analyses stratified by sex).

We next performed multivariable analyses which included age at immunotherapy initiation, sex, smoking status, ECOG performance status at immunotherapy initiation, and PWL (sex was not included in the analyses stratified by sex) (Fig. 2). We again observed no association between BMI and PFS and OS. Among those treated with first-line monotherapy, underweight patients were at increased risk of disease progression (HR: 3.50, 95% CI 1.45–8.43, *P* = 0.005) and death (HR: 2.48, 95% CI 0.99–6.22, *P* = 0.054) compared to normal weight patients. This was not observed in the other treatment groups. In addition, underweight males were overall at increased risk of death (HR: 5.35, 95% CI 2.42–11.81, *P* < 0.0001) and disease progression (HR: 9.08, 95% CI 4.19–16.69, *P* < 0.0001) compared to normal weight males. A similar relationship between BMI and outcome was not observed for females. Additional adjustment for PD-L1 status and creatinine level did not alter these results (data not shown).

### Pretreatment weight loss was associated with worse outcome in both sexes

**Figure 3:**
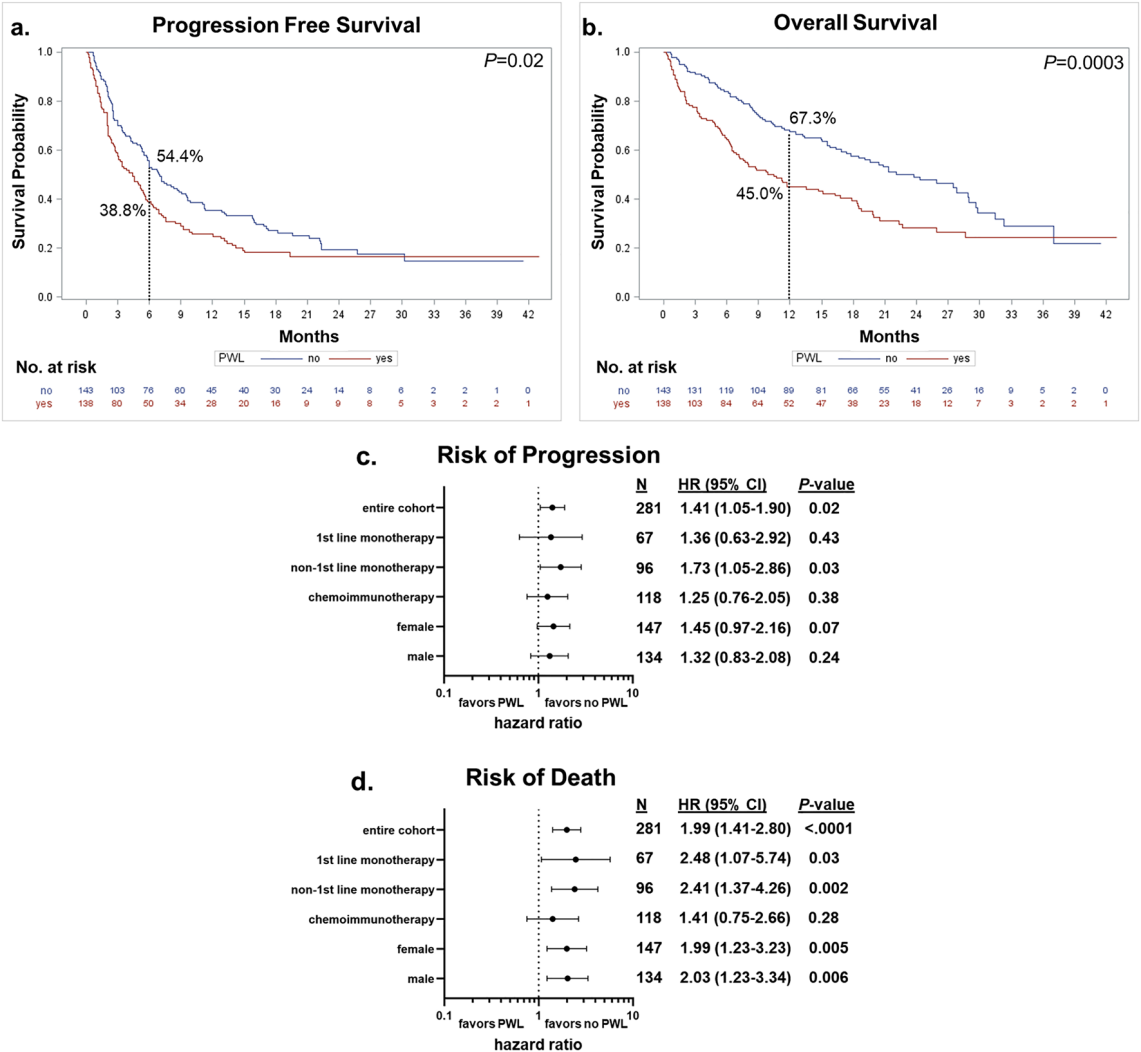
Pretreatment weight loss and patient outcomes. (**a**) Kaplan–Meier progression free survival (PFS) estimates by pretreatment weight loss (PWL) status. Six-month PFS rate is indicated. (**b**) Kaplan–Meier overall survival (OS) estimates by PWL status. Twelve-month OS rate is indicated. Curves were compared using log-rank tests. (**c**) and (**d**) are Forest plots reporting results from the multivariable analyses of the effect of PWL on risk of disease progression and death, respectively. The reference group is “no PWL”, adjusted hazard ratios (HRs), corresponding 95% confidence intervals (CIs) and *P* values are shown. Variables included in the Cox proportional hazards model: age at immunotherapy initiation, sex (female, male), BMI at immunotherapy initiation (underweight, normal, overweight, obese), smoking status (never, former, current), ECOG performance status at immunotherapy initiation (0–1, 2+, unknown) (sex was not included in the analyses stratified by sex).

To assess the effect of PWL on patient outcomes, we limited our study population to those for whom this information was available, N = 281. The distributions of the baseline characteristics in this subset were similar to those in the total study population (see Supplemental Table 1). Selected demographic and clinical characteristics stratified by PWL status are presented in Table 4. Although the disease control rate was lower (54.4%) in the patients with PWL compared to those without (65.0%), this difference was not statistically significant (*P* = 0.07). Patients with PWL were significantly more often underweight or normal weight at immunotherapy treatment initiation (60.9% *vs*. 29.4%). They also more often had worse performance status (23.2% *vs*. 11.2% ECOG 2 +). Kaplan–Meier curves for PFS and OS by PWL status are presented in Fig. 3a,b. Both PFS and OS were significantly worse for patients with PWL compared to those without (*P* = 0.02 and *P* = 0.0003, respectively). Median PFS for those with and without PWL were 4.4 months (95% CI 3.0–5.6) and 7.0 months (95% CI 5.5–9.5), respectively; median OS was 10.5 months (95% CI 6.9–18.3) and 23.7 months (95% CI 17.4–29.0), respectively.

**Table 4:** Characteristics by pretreatment weight loss status (Yes/No; N_total_ = 281).

N(%)	Yes (N = 138)	No (N = 143)	*P*-value^a^
Age at IT^b^ initiation in yrs			0.79
Mean (± sd)	69.4 (± 10.0)	69.0 (± 10.0)	
Sex, Female	77 (55.8)	70 (49.0)	0.25
Smoking status			0.40
Never	9 (6.5)	10 (7.0)	
Former	67 (48.6)	80 (55.9)	
Current	62 (44.9)	53 (37.1)	
Performance status (ECOG)			0.02
0–1	93 (67.4)	117 (81.8)	
2 +	32 (23.2)	16 (11.2)	
Unknown	13 (9.4)	10 (7.0)	
PD-L1 status			0.72
< 1%	54 (39.1)	57 (39.9)	
1–50%	30 (21.7)	38 (26.6)	
> 50%	38 (27.5)	33 (23.1)	
Unknown	16 (11.6)	15 (10.5)	
Body mass index at IT initiation in categories			< .0001
Underweight (< 18.5 kg/m^2^)	19 (13.8)	4 (2.8)	
Normal weight (18.5–24.9 kg/m^2^)	65 (47.1)	38 (26.6)	
Overweight (25–29.9 kg/m^2^)	35 (25.4)	55 (38.5)	
Obese (≥ 30 kg/m^2^)	19 (13.8)	46 (32.2)	
Treatment line			0.87
1st line monotherapy	33 (23.9)	34 (23.8)	
Non-1st line monotherapy	49 (35.5)	47 (32.9)	
Chemoimmunotherapy	56 (40.58)	62 (43.4)	
Best response			0.07
Disease control (CR, PR, or SD)^c^	75 (54.4)	93 (65.0)	
Progressive disease (PD)	63 (45.7)	50 (35.0)	
Immune-related adverse events			0.35
Yes	42 (30.4)	51 (35.7)	
No	96 (69.6)	92 (64.3)	

^a^*P* value from chi-square test for categorical variables and from Wilcoxon–Mann–Whitney test for continuous variables.

^b^IT: immunotherapy.

^c^CR, complete response; PR, partial response; SD, stable disease.

Stratified by treatment line, PFS was significantly worse for those with PWL compared to those without in the non-first line monotherapy group only (*P* = 0.046, 6-month PFS rate: 22.0% *vs*. 46.5%; Supplemental Fig. 2a). OS was significantly worse for those with PWL in both the first-line and non-first line groups (*P* = 0.03, 12-month OS rate: 38.8% *vs*. 67.5%, and *P* = 0.005, 38.7% *vs*. 68.1%, respectively; Supplemental Fig. 2b). Stratified by sex, those who experienced PWL had a significantly increased risk of disease progression (*P* = 0.03; 6-month PFS rate: 35.6% *vs*. 54.4%) and risk of death (*P* = 0.001; 12-month OS rate: 37.7% *vs*. 66.5%) compared to those with stable weight among males, and a significant increased risk of death among females (*P* = 0.03; 12-month OS rate: 50.6% *vs.* 68.2%) (Supplemental Fig. 2c-d).

We next performed multivariable analyses which are reported in Fig. 3c,d and include age at immunotherapy initiation, BMI at immunotherapy initiation, sex (except when stratified by sex), smoking status, and ECOG performance status at immunotherapy initiation. Worse outcomes for those with PWL was again observed. Compared to stable weight, PWL was associated with increased risk of disease progression and death (HR: 1.41, 95% CI 1.05–1.90, *P* = 0.02 and HR: 1.99, 95% CI 1.41–2.80, *P* < 0.0001, respectively). Similarly, stratified by treatment line, risk of disease progression was significantly higher for those with PWL among those receiving non-first line monotherapy (HR: 1.73, 95% CI 1.05–2.86, *P* = 0.03) while risk of death was significantly higher among those receiving first-line and non-first line monotherapy (HR: 2.48, 95% CI 1.07–5.74, *P* = 0.03, and HR: 2.41, 95% CI 1.37–4.26, *P* = 0.002, respectively). Stratified by sex, PWL significantly increased risk of death among both males and females (HR: 2.03, 95% CI 1.23–3.34, *P* = 0.006, and HR: 1.99, 95% CI 1.23–3.23, *P* = 0.005, respectively) but was not associated with risk of progression. Additional adjustment for PD-L1 status and creatinine level did not alter these results (data not shown).

## Discussion

ICIs alone or chemoimmunotherapy are the current standard of care for metastatic NSCLC without targetable mutations^2^. Unfortunately, many patients do not respond and most tumors are ultimately refractory to treatment. Better identification of patients likely to benefit from immunotherapy is clearly needed. Recent studies report conflicting findings on the influence of sex and BMI on response to ICIs^5–11, 25–30, 32–34^, while emerging evidence suggests a potential link between cachexia status and poor response to ICIs^12–15, 18^. In this retrospective study, we evaluated the effects of sex, BMI and PWL on outcomes in metastatic NSCLC patients treated with ICIs as part of standard of care at our institution.

In our cohort, females had overall a significantly lower risk of death compared to males. In analyses stratified by treatment line, this survival benefit was significant only in those treated with first-line monotherapy. These findings are in line with some studies, but not with all. While there is clear sex-based dimorphism in the innate and adaptive immune systems^24^, there are conflicting data regarding sex differences in ICI efficacy in NSCLC^27–30, 32–34^. Historically, female sex has been associated with improved outcomes in NSCLC across all stages, even after controlling for variables such as *EGFR* mutation status^35, 36^. A seminal meta-analysis of advanced NSCLC randomized controlled trials of PD-(L)1 inhibitors showed that compared to chemotherapy alone, chemoimmunotherapy benefits females more than males whereas ICI monotherapy benefits males more than females^27^. Subsequent meta-analyses could not replicate these findings, reporting no sex difference in response to either treatment group, but may have introduced heterogeneity by including CTLA-4 inhibitor, non-first line therapy and adjuvant trials^30, 32^. Of note, these studies had no access to patient level data and could not directly compare males to females. Utilizing patient-level data, larger observational studies have reported that females have improved survival compared to males treated with chemoimmunotherapy and second-line ICI monotherapy^29, 34^, while smaller studies found no difference in survival between the sexes^28, 33^. Our findings may reflect the effect of a better prognosis for female NSCLC patients regardless of treatment modality, despite the relative benefit males may derive from ICI monotherapy compared to chemotherapy.

Female patients in our cohort more often experienced irAEs, particularly mild adverse events not requiring steroids. This is in line with what other retrospective studies have reported^37^, although a more recent study suggested there are minimal sex-associated differences in irAEs among cancer patients^38^. Presence of irAEs is known to be associated with improved ICI efficacy and survival^39^. Possible explanations for this relationship include shared antigens between the tumor and inflamed organ, “off-target” effects of T-cell activation, or certain microbes in the gut microbiome that provoke both response and colitis^39^.

BMI was overall not significantly associated with outcome in our study population. Additional adjustment for PD-L1 status did not change these results. In stratified analyses, being underweight was associated with worse outcomes compared to being normal weight, but only among males and those treated with first-line monotherapy. There is substantial evidence that adipose tissue in obese individuals alters the TIME as well as preclinical evidence in multiple murine models and clinical data showing that obesity modulates response to ICIs^40^. In NSCLC specifically, obesity has been reported to be associated with improved survival in PD-L1 positive patients treated with ICI monotherapy^5, 8^. However, a separate study reported no significant differences in outcomes between PD-L1 positive patients with BMI ≥ 22* versus* BMI < 22^9^. Although our cohort includes a substantial proportion of obese patients (24.5%), we did not observe a survival benefit in obese patients; however, the number of PD-L1 positive ICI-monotherapy treated patients in our study may have been insufficient to detect a benefit of obesity. In addition, in previous studies, underweight patients were either not included in the analysis^5^, placed in a “low BMI group” that included all patients with BMI < 22^9^ or BMI < 25^7^, or used as the comparator to the normal, overweight and obese groups^9^. In support of our findings, Cortellini et al. reported that underweight NSCLC patients treated with first-line pembrolizumab did worse than normal weight patients but this difference was not significant^8^. Similarly, Ichihara et al*.* found that normal weight, overweight and obese and NSCLC patients treated with non-first line ICIs had longer PFS and OS compared with underweight patients^9^. A recent meta-analyses showed that when stratified by sex, the effect of BMI on ICI efficacy was restricted to males^41^. Together, these findings suggest that being underweight could impair immunotherapy efficacy in a sex-specific manner.

While PWL was not significantly associated with disease control, it was an independent predictor of worse OS, a finding that seems to be driven by the first-line and non-first line monotherapy groups and held true for both males and females. Cachexia is a syndrome of anorexia, muscle wasting, and loss of body mass that commonly portends a poor prognosis in cancer patients^42^. Our findings are consistent with other studies reporting a negative association of cachexia with ICI outcomes, including a recent prospective study that investigated multiple cachexia-related inflammatory, body composition, and nutritional measures^13–17, 43^.

Strategies to counteract underweight status, PWL and cancer cachexia should be considered to improve the efficacy of ICIs. There are currently no approved treatments beyond nutritional and exercise recommendations, but several clinical trials testing pharmacologic agents against effects of cachexia are underway. Cachexia is associated with, and may represent the downstream sequelae of a proinflammatory state with elevation in TNF-α, IL-6, IL-1, and IFNγ^44^. These proinflammatory cytokines generate an immunosuppressive environment, which may decrease the efficacy of immunotherapy^45^. When combined with ICIs, TNF-α blockade improved efficacy in murine models of melanoma, lung cancer and colon cancer^46, 47^. A recent phase Ib trial of nivolumab and ipilimumab in combination with TNF-α inhibitors in patients with advanced melanoma found the combination to be safe with promising clinical responses^48^. Another promising potential target is growth differentiation factor (GDF15), a stress-response cytokine secreted by tumor and immune cells which activates glial cell line-derived neurotrophic factor family receptor a-like (GFRAL) to regulate body weight^49^. Elevated plasma GDF15 levels are found in lung cancer patients with unintentional weight loss and is a predictor of poor prognosis^50^. In a preclinical mouse model of cancer-induced cachexia, GDF15 neutralization alleviated weight loss and restored muscle mass and function^51^. The GDF15 targeting agent, ponsegromab, is currently being evaluated in a phase 2 clinical trial of cancer patients with cachexia and elevated GDF15 (NCT05546476) and a separate agent CTL-002 is being tested in combination with ICIs in patients with advanced cancer (NCT04725474).

Our stratified analyses suggest that patient sex, BMI status and PWL status do not independently affect outcome of treatment with chemoimmunotherapy. These three factors do appear to affect outcome of first-line monotherapy, and PWL status may affect outcome of non-first line monotherapy. There are mixed results reported on PWL and chemoimmunotherapy outcomes. Our results are in line with a previous study reporting that BMI does not affect outcomes in chemoimmunotherapy-treated patients^10^, however incongruent with other studies reporting that PWL^17^ and male sex adversely impacts outcomes^29^. In one study, PWL was associated with worse PFS in the chemoimmunotherapy group, with no difference in OS; the worse PFS was not observed when evaluating only the PD-L1 > 50% population^16^. When given as combination therapy, chemotherapy can sensitize tumors to immunotherapy through a variety of mechanisms^52^. It is possible that administering chemotherapy with immunotherapy, perhaps with a limited course as described in the CheckMate 9LA trial^53^, could help overcome immunosuppressive effects of male sex, being underweight or PWL and improve outcomes for patients with these characteristics. Of note, almost 41% of our patients with PWL received chemoimmunotherapy suggesting that this would be feasible in that patient population. Ultimately, the decision between initiating chemoimmunotherapy or ICI monotherapy as first-line therapy in PD-L1 expressing patients remains complex and must be individualized. Randomized prospective studies are needed to further delineate the benefits of each treatment modality.

An important strength of this study is that we used real-world individual level data and were able to collect detailed information on pretreatment weight for the majority of the included patients. However, due to its retrospective nature, information on pretreatment weight and also PD-L1 status was not available for all patients. In addition, response and progression were determined based on radiographic reports and clinical information available in the electronic medical record. As such, a retrospective imaging analysis was not performed utilizing RECIST or iRECIST criteria given lack of access to radiographic images. Sample size limitations, in particular in the stratified analyses, may have precluded the detection of statistically significant findings. It should also be noted that our study population was primarily white which may limit generalizability of the results.

To conclude, this real-world analysis suggests that male sex, being underweight, and PWL negatively impact ICI efficacy in NSCLC patients. Further studies are needed to confirm these findings and to explore ways to counteract being underweight and PWL prior to ICI initiation.

### Supplementary Information

Supplementary Figures.

Supplementary Table 1.

## Data Availability

The datasets generated during and/or analyzed during the current study are available from the corresponding authors on reasonable request.
