# Supplementary material for: Male sex and pretreatment weight loss are associated with poor outcome in patients with advanced non-small cell lung cancer treated with immunotherapy: a retrospective study
Source: Sci Rep. 2023 Oct 9;13:17047. doi: 10.1038/s41598-023-43866-5 (PMC10562448; doi:10.1038/s41598-023-43866-5)
Supplement: Supplementary file 1 — Supplementary Figures. [file 41598_2023_43866_MOESM1_ESM.pdf]

Male sex and pretreatment weight loss are associated with poor outcome in patients with advanced non-small cell lung cancer treated with immunotherapy: a retrospective study

Jingxiao Jin[1], Jacqueline Visina[2], Timothy F. Burns[1,3,4], Brenda Diergaarde[3,5*], and Laura P. Stabile[3,4*]

[1] Department of Medicine, Division of Hematology-Oncology, University of Pittsburgh, Pittsburgh, PA, USA

[2] Department of Internal Medicine, Vanderbilt University Medical Center, Nashville, TN, USA

[3] UPMC Hillman Cancer Center, Pittsburgh, PA, USA

[4] Department of Pharmacology & Chemical Biology, University of Pittsburgh, Pittsburgh, PA

[5] Department of Human Genetics, School of Public Health, University of Pittsburgh, Pittsburgh, PA, USA

* Corresponding Authors

Corresponding Authors: Laura P. Stabile, UPMC Hillman Cancer Center, Hillman Cancer Center Research Pavilion, Office: Suite 2.18d Lab: 2.7, 5117 Centre Avenue, Pittsburgh, PA 15213-1863; Phone: 412-623-2015; Email: stabilela@upmc.edu; ORCID ID 0000-0002-1822-2707

Brenda Diergaarde, UPMC Hillman Cancer Center, UPMC Cancer Pavilion, Suite 4C, Office #467, 5150 Centre Avenue, Pittsburgh, PA 15213-1863. Phone: 412-623-5891; Email: diergaardeb@upmc.edu; ORCID ID 0000-0002-3578-6547

Supplemental Figure 1

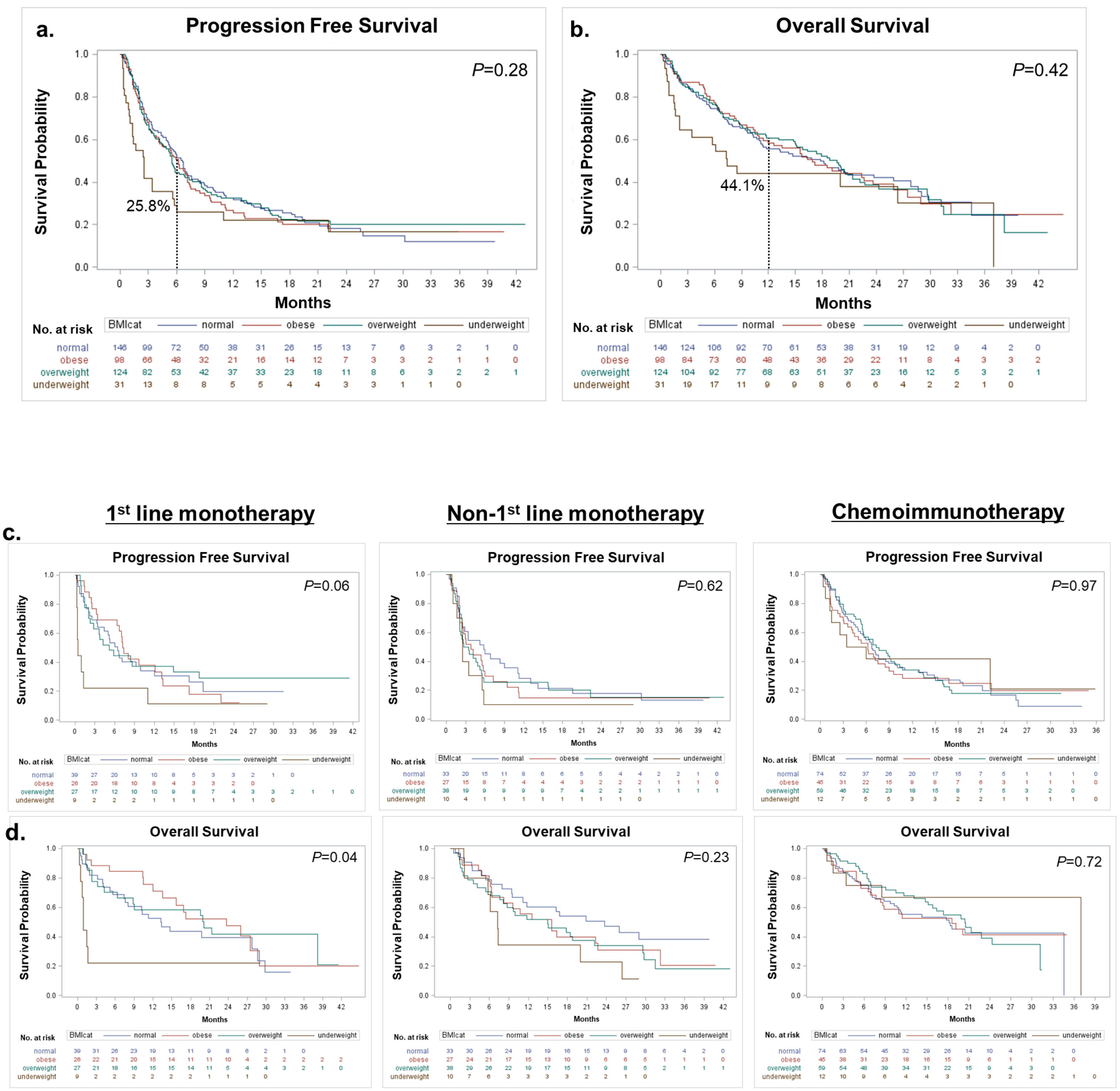

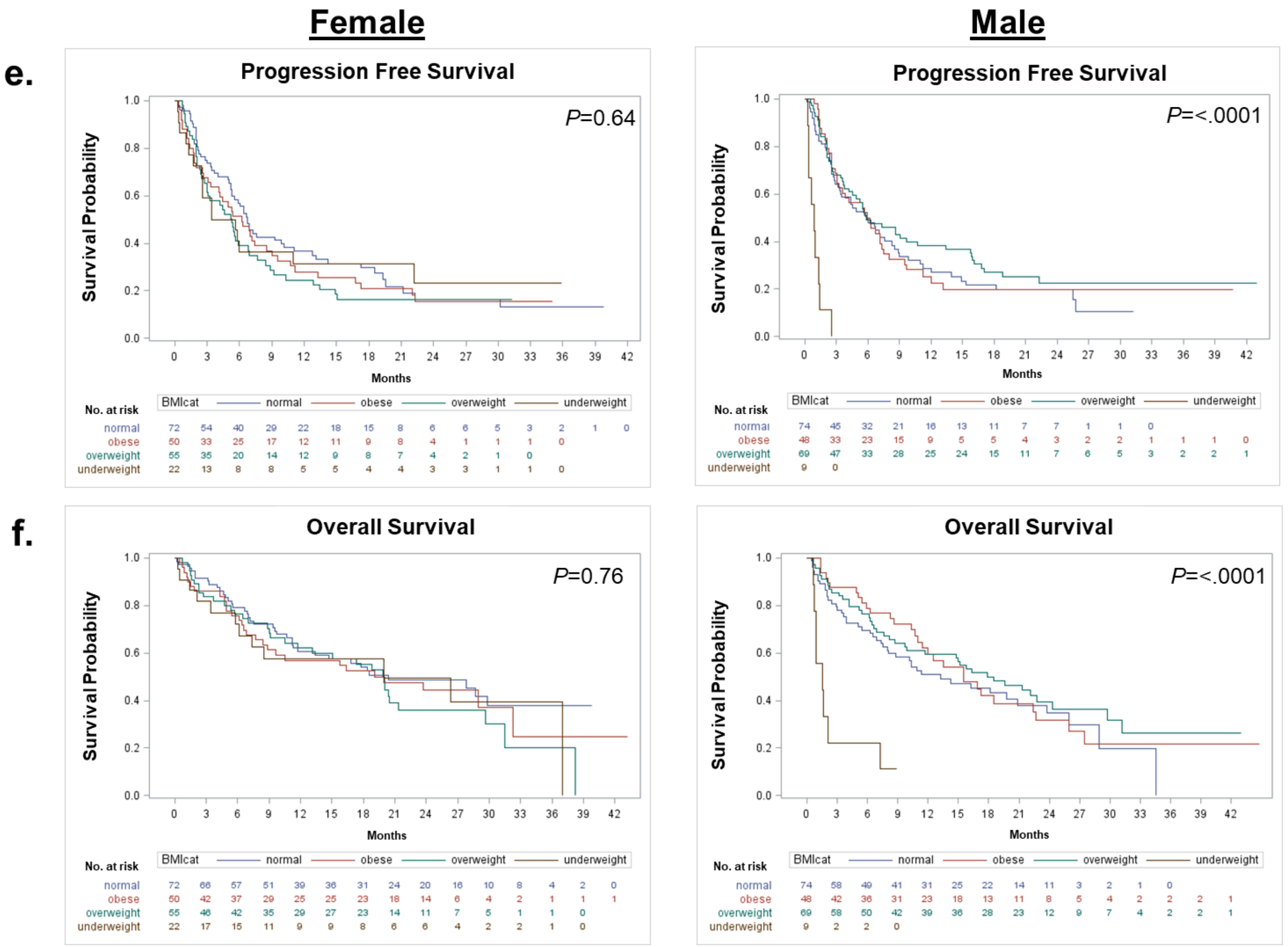

Supplemental Figure 1. (a) Kaplan-Meier progression free survival (PFS) estimates by body mass index (BMI) category. Six-month PFS rate is indicated for the underweight group. (b) Kaplan-Meier overall survival (OS) estimates by BMI category. Twelve-month OS rate is indicated for the underweight group). (c) Kaplan-Meier PFS estimates by BMI category separately for each of the three treatment groups. (d) Kaplan-Meier OS estimates by BMI category separately for each of the three treatment groups. (e) Kaplan-Meier PFS estimates by BMI category separately for females and males. (f) Kaplan-Meier OS estimates by BMI category separately for females and males. Curves were compared using log-rank tests.

Supplemental Figure 2

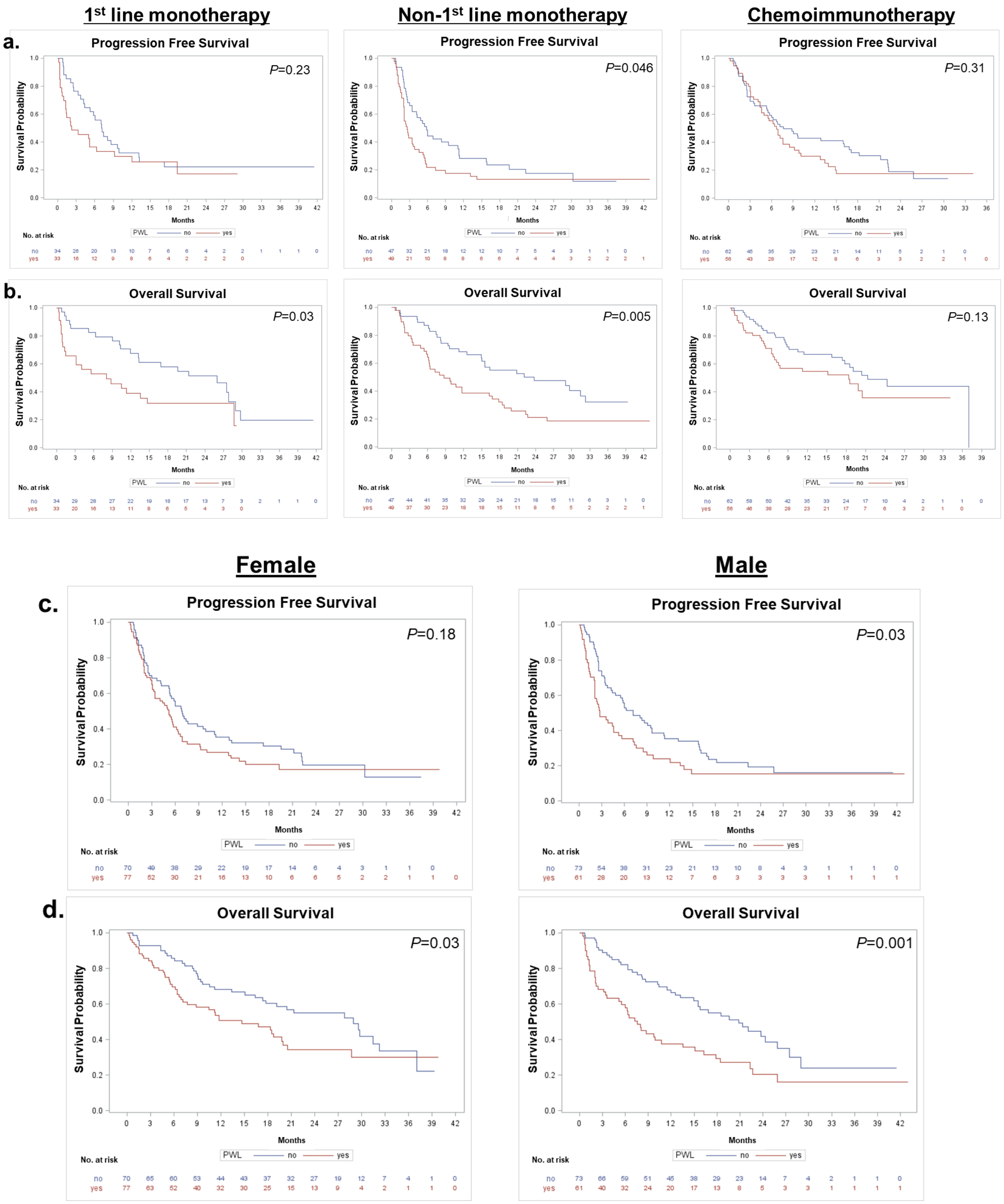

Supplemental Figure 2. (a) Kaplan-Meier progression free survival (PFS) estimates by pretreatment weight loss (PWL) status separately for each of the three treatment groups. (b) Kaplan-Meier overall survival (OS) estimates by PWL status separately for each of the three treatment groups. (c) Kaplan-Meier PFS estimates by PWL status separately for females and males. (d) Kaplan-Meier OS estimates by PWL status separately for females and males. Curves were compared using log-rank tests.
