# Supplementary material for: Male sex and pretreatment weight loss are associated with poor outcome in patients with advanced non-small cell lung cancer treated with immunotherapy: a retrospective study
Source: Sci Rep. 2023 Oct 9;13:17047. doi: 10.1038/s41598-023-43866-5 (PMC10562448; doi:10.1038/s41598-023-43866-5)
Supplement: Supplementary file 2 — Supplementary Table 1. [file 41598_2023_43866_MOESM2_ESM.docx]

**Supplemental Table 1:** Total study population *versus* patients with available pretreatment weight loss information

| N (%) | Total study population, N=399^1^ | Patients with PWL information, N=281^1^ |
| --- | --- | --- |
| **Age at IT**^2^ **initiation in yrs**  mean (± sd)  range | 68.0 (± 9.9)  (33.1-92.6) | 69.2 (± 10.0)  (33.1-92.6) |
| **Sex**, **female** | 199 (49.9) | 147 (52.3) |
| **Race**  White  Black/African American  American Indian/Alaska Native  Asian  Unknown | 348 (87.2)  36 (9.0)  1 (0.3)  6 (1.5)  8 (2.0) | 248 (88.3)  26 (9.3)  0  3 (1.1)  4 (1.4) |
| **Smoking status**  Never  Former  Current | 23 (5.8)  188 (47.4)  186 (46.9) | 19 (6.8)  147 (52.3)  115 (40.9) |
| **Performance status (ECOG)**  0-1  2+  Unknown | 301 (75.4)  63 (15.8)  35 (8.8) | 210 (74.7)  48 (17.1)  23 (8.2) |
| **Serum creatinine (mg/dL), mean (± sd)** | 0.94 (± 0.48) | 0.94 (± 0.48) |
| **Albumin (g/dL), mean (± sd)** | 3.54 (± 0.57) | 3.56 (± 0.52) |
| **Neutrophil-lymphocyte ratio, mean (± sd)** | 8.3 (± 10.6) | 8.4 (± 11.0) |
| **Body mass index at IT initiation in kg/m^2^**  mean (±sd)  range | 26.4 (± 6.2)  (13.4-49.4) | 26.2 (± 6.0)  (13.4-44.1) |
| **Body mass index at IT initiation in categories**  underweight (<18.5 kg/m^2^)  normal weight (18.5-24.9 kg/m^2^)  overweight (25-29.9 kg/m^2^)  obese (≥30 kg/m^2^) | 31 (7.8%)  146 (36.6%)  124 (31.1%)  98 (24.5%) | 23 (8.2)  103 (36.7)  90 (32.0)  65 (23.1) |
| **Histology**  Adenocarcinoma  Squamous cell carcinoma  Other | 263 (65.9)  77 (19.3)  59 (14.8) | 187 (66.5)  57 (20.3)  37 (13.2) |
| **Metastatic stage**  M1a  M1b  M1c | 130 (32.6)  60 (15.0)  209 (52.4) | 103 (36.7)  40 (14.2)  138 (49.1) |
| **PD-L1 status**  <1%  1-50%  >50%  Unknown | 146 (36.6)  94 (23.6)  117 (29.3)  42 (10.5) | 111 (39.5)  68 (24.2)  71 (25.3)  31 (11.0) |
| **Immunotherapy received**  Pembrolizumab  Atezolizumab  Nivolumab^3^ | 283 (70.9)  71 (17.8)  45 (11.3) | 181 (64.4)  57 (20.3)  43 (15.3) |
| **Treatment line**  1^st^ line monotherapy  Non-1^st^ line monotherapy  Chemoimmunotherapy | 101 (25.3)  108 (27.1)  190 (47.6) | 67 (23.8)  96 (34.2)  118 (42.0) |
| **Best response**  Disease control (CR, PR, or SD)^4^  Progressive disease (PD) | 242 (60.7)  157 (39.4) | 168 (59.8)  113 (40.2) |
| **Immune-related adverse events (irAEs)**  No AE  AE mild  AE steroids^5^  AE limiting^6^ | 266 (66.7)  54 (13.5)  30 (7.5)  49 (12.3) | 188 (66.9)  40 (14.2)  21 (7.5)  32 (11.4) |
| **Alive at last follow up** | 165 (41.4) | 114 (40.6) |

^1^ Does not always add up to N=399 or N=281 due to missing information.

^2^ IT: immunotherapy.

^3^ Includes patients who received nivolumab plus ipilimumab, N=3 in the total study population and N=3 in the subset of patients with PWL information.

^4^ CR: complete response; PR: partial response; SD: stable disease.

^5^ AE requiring steroid treatment but not therapy limiting.

^6^ AE that was therapy limiting.
